# Social determinants of malaria in an endemic area of Indonesia

**DOI:** 10.1186/s12936-019-2760-8

**Published:** 2019-04-12

**Authors:** Hamzah Hasyim, Pat Dale, David A. Groneberg, Ulrich Kuch, Ruth Müller

**Affiliations:** 1 Institute for Occupational Medicine, Social Medicine and Environmental Medicine, Faculty of Medicine, Goethe University, Frankfurt Am Main, Germany; 2 Faculty of Public Health, Sriwijaya University, Indralaya, South Sumatra Indonesia; 3 Environmental Futures Research Institute (EFRI), School of Environment & Science, Griffith University, Nathan, QLD Australia; 4 Unit of Entomology, Institute of Tropical Medicine, 2000 Antwerp, Belgium

**Keywords:** Multivariable analysis, Malaria prevalence, Social health determinants, Social epidemiology, Community health services

## Abstract

**Background:**

Malaria is an increasing concern in Indonesia. Socio-demographic factors were found to strongly influence malaria prevalence. This research aimed to explore the associations between socio-demographic factors and malaria prevalence in Indonesia.

**Methods:**

The study used a cross-sectional design and analysed relationships among the explanatory variables of malaria prevalence in five endemic provinces using multivariable logistic regression.

**Results:**

The analysis of baseline socio-demographic data revealed the following independent risk variables related to malaria prevalence: gender, age, occupation, knowledge of the availability of healthcare services, measures taken to protect from mosquito bites, and housing condition of study participants. Multivariable analysis showed that participants who were unaware of the availability of health facilities were 4.2 times more likely to have malaria than those who were aware of the health facilities (adjusted odds ratio = 4.18; 95% CI 1.52–11.45; *P* = 0.005).

**Conclusions:**

Factors that can be managed and would favour malaria elimination include a range of prevention behaviours at the individual level and using the networks at the community level of primary healthcare centres. This study suggests that improving the availability of a variety of health facilities in endemic areas, information about their services, and access to these is essential.

**Electronic supplementary material:**

The online version of this article (10.1186/s12936-019-2760-8) contains supplementary material, which is available to authorized users.

## Background

Malaria is a significant public health problem especially in developing countries including Indonesia [1]. Research has shown an enhanced interest in the social aspects of the epidemiology of malaria prevalence [2]. Socio-demographic, environmental, economic, cultural and behavioural factors determine the frequency, severity and outcome of malaria infection [3, 4]. Based on the Indonesian basic health research (*Riskesdas*) the prevalence of malaria in 2013 was 6.0%. The distribution of the disease is focussed on eastern Indonesia [5, 6]. Of 497 districts/municipalities of Indonesia, 54% are endemic areas for malaria. The Ministry of Health (MoH) strategy plan for malaria morbidity targeted an Annual Parasite Incidence (API) of < 1 per 1000 population at risk by 2015 [7]. Nationally, malaria morbidity decreased from 4.1 per 1000 people in 2005 to 0.85 per 1000 by 2015 [7]. Reducing the anopheline vectors has been the subject of many meetings and public health initiatives for decades [8]. It has been proposed to eliminate malaria from Indonesia by 2030, with a variety of agendas particularly for endemic areas [9]. As the burden of malaria is very complicated, its elimination, implemented through an integrated approach, has become an integral part of national development [10]. This study attempts to identify socio-demographic factors that are related to malaria prevalence in Indonesia, such as the characteristics of participants, knowledge of the accessibility and utilization of health services, environmental health factors including personal measures to protect from mosquito bites, and the condition of housing structures.

## Methods

### Study area

**Fig. 1:**
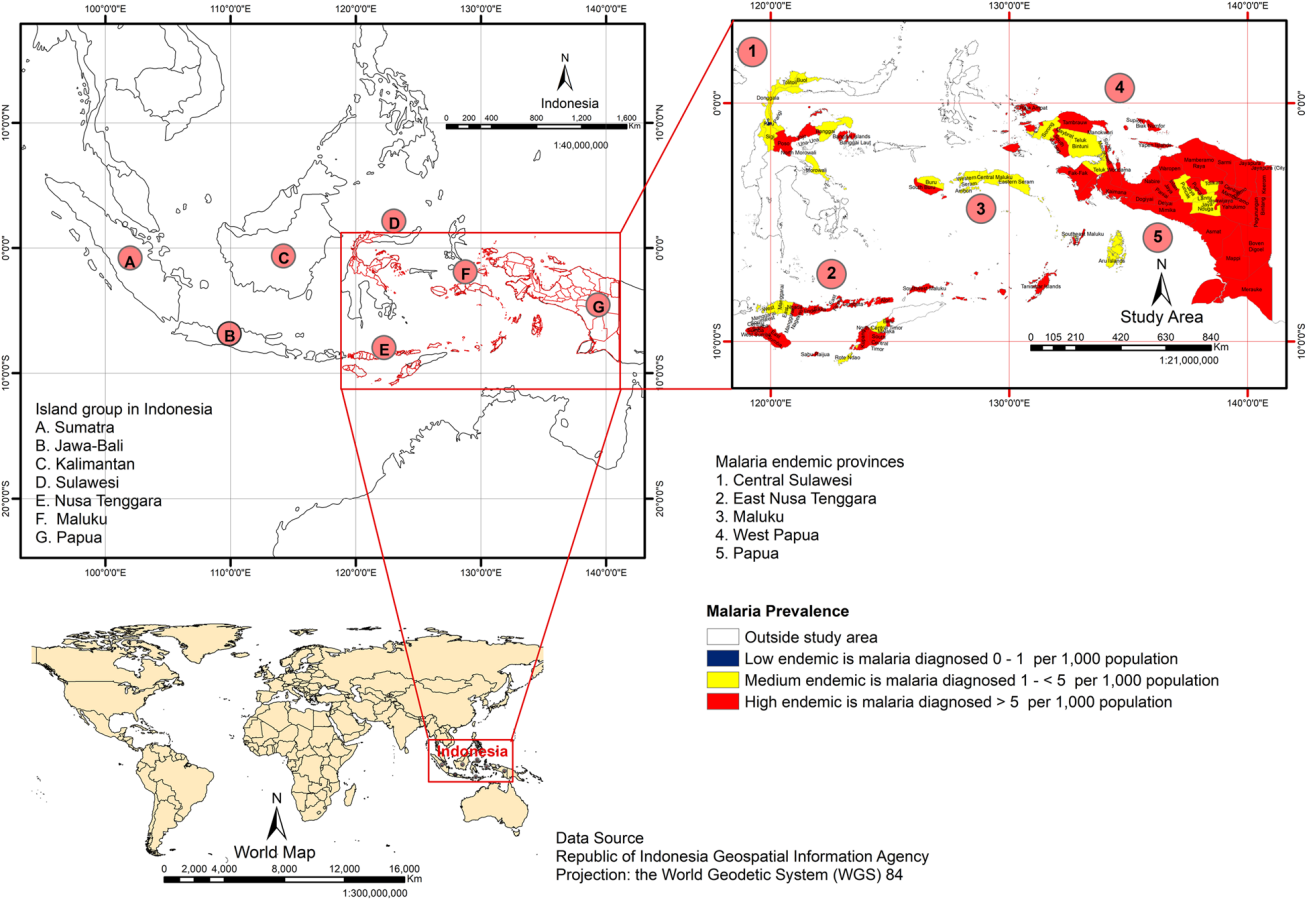
Map of the study areas

The study area covered five out of 33 provinces of Indonesia (83 out of 497 districts and cities in 2013): Central Sulawesi, East Nusa Tenggara, Maluku, Papua, and West Papua Provinces (Fig. 1). These provinces were selected because they had been shown to be highly endemic for malaria both in the 2007 and 2013 basic health research of Indonesia [5, 6]. A “highly malaria endemic” area was defined as having > 5 cases of malaria diagnosed per 1000 population and year which is consistent with the API classification by the MoH of Indonesia. The software ArcGIS 10.3.1 was used for mapping, processing, analysis, and visualization of the data set, and WGS84 was used as the reference coordinate system.

### Research design

**Fig. 2:**
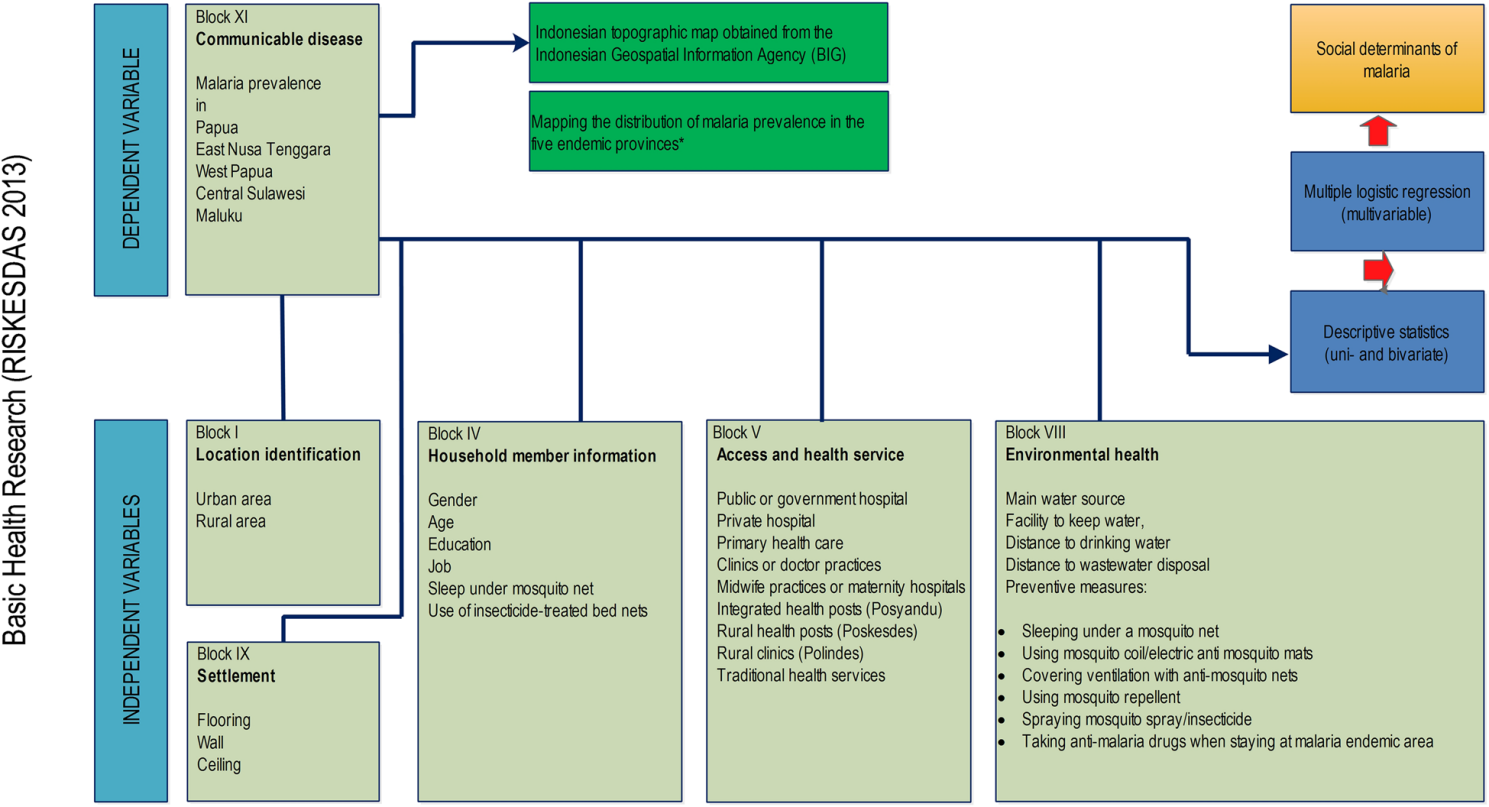
Framework determinants of malaria among participants in the selected area

The design of the Indonesian basic health research, which is called Riskesdas, is a descriptive cross-sectional survey to describe public health problems throughout Indonesia [6]. Figure 2 shows its framework for malaria research. The sample comprised 130,585 participants who represented the population in five highly malaria-endemic provinces.

### Research variables

The dependent variable was malaria prevalence and is binary, that is, whether malaria was present or absent. The definition of disease used was diagnosis of the participants (D) with malaria by a physician or professional health worker (Additional file 1: Appendix S1). The data were obtained from a retrospective assessment by health surveyors using a standardised questionnaire. Participants who claimed to have never been diagnosed with malaria were asked whether they had suffered from the specific signs and clinical symptoms of the disease. The term “diagnosed/clinical symptoms” means that the prevalence of illness was based on the diagnosis by a physician or health worker in a health centre or based on the signs and symptoms experienced and reported by participants. The report referred to the disease information collected from interviews using questionnaires and clinically measured interviews [5, 6]. The dependent variable, malaria prevalence, was summarized as a binary variable whose value was one if health experts assessed a participant as having had malaria within the past month [5, 6]. In general, rapid diagnostic tests (RDTs) and microscopy were used to diagnose the disease, but the surveyor did not examine for malaria infection [5, 6].

The explanatory variables consisted of several socio-demographic factors that could affect malaria prevalence including the characteristics of participants, the availability of healthcare services, environmental sanitation including behaviour to prevent mosquito bites, and settlement (Fig. 2, Additional file 1: Appendix S1). These variables were grouped into blocks based on the questionnaire: block I—location identification or household information; block IV—household member information includes sex, age group (year), education and job (occupation), use of bed nets for sleeping and net insecticide; block V—knowledge of available healthcare facilities; block VIII—environmental health including prevention measures against malaria; and block IX—settlement (condition of housing structure). These were the criteria for environmental health in Riskesdas 2013 (joint monitoring programme World Health Organization–the United Nations Children’s Fund criteria). Using logistic regression, the independent variables were standardized and modified by considering the survey design [11]. Variables that were transformed into categorical variables were: knowledge of available healthcare facilities, environmental sanitation, prevention measures, and condition of housing structure. All variables were coded as binary dummy variables coded 0 as referent category and coded 1 for a response category of an explanatory variable. Stata was used for data management and analysis [12, Additional file 1: Appendix S1].

### Descriptive analysis

The descriptive analysis aimed to identify the characteristics of the independent variables in relation to the dependent variable, malaria prevalence. The variables are summarized in Table 1 and show the baseline socio-demographic characteristics of study participants. The magnitude of risk for having malaria was assessed from the calculated odds ratio (OR) and AOR (bi- and multivariable logistic regression test). If an OR was higher than one, the likelihood of contracting malaria was increased.

**Table 1:** Univariate and bivariate analysis of baseline socio–demographic characteristics of participants

Research variables	n = 130,585	95% CI (lb–ub)^a^	OR; 95% CI (lb–ub)^b^	P-value
Malaria				
No	116,073	89.90 (89.15–90.6)		
Yes	14,512	10.10 (9.40–10.85)		
Independent variables				
Location				
Urban	37,389	25.60 (23.07–28.31)		
Rural	93,196	74.40 (71.69–76.93)	0.91 (0.76–1.09)	0.305
Socio–demographic characteristics				
Gender				
Male	64,796	51.08 (50.76–51.40)		
Female	65,789	48.92 (48.60–49.24)	0.90 (0.85–0.94)	0.000
Age of participants in years				
0–4	10,109	8.52 (8.27–8.78)		
5–14	33,378	26.06 (25.60–26.52)	1.32 (1.18–1.49)	0.000
15–24	17,623	15.49 (15.09–15.90)	1.29 (1.14–1.47)	0.000
25–34	19,420	17.09 (16.70–17.47)	1.45 (1.29–1.64)	0.000
35–44	19,604	13.77 (13.47–14.09)	1.58 (1.39–1.80)	0.000
45–54	14,170	8.90 (8.65–9.17)	1.42 (1.24–1.62)	0.000
55–64	8312	4.84 (4.64–5.05)	1.27 (1.09–1.50)	0.003
65–74	3927	2.38 (2.25–2.52)	1.14 (0.96–1.36)	0.147
> 75	4042	2.94 (2.81–3.08)	1.33 (1.12–1.58)	0.001
Education				
Participants considered as higher educated	5935	4.193 (3.853–4.562)		
Participants who had not completed high school education	94,644	72.08 (71.33–72.83)	0.99 (0.83–1.18)	0.878
Participants under 10 years or in preschool	30,006	23.72 (22.93–24.54)	0.84 (0.69–1.03)	0.092
Job (occupation)				
Participants who were not working	77,533	60.12 (59.42–60.82)		
Participants who were working	53,052	39.88 (39.18–40.58)	1.20 (1.12–1.27)	0.000
Use of mosquito nets				
Participants who used mosquito nets at night	61,779	46.19 (44.12–48.27)		
Participants who did not use mosquito nets at night	68,806	53.81 (51.73–55.88)	1.09 (0.97–1.23)	0.153
Use of i insecticide–treated mosquito nets				
Yes	32,150	23.26 (21.73–24.85)		
No	27,510	21.49 (20.03–23.02)	0.90 (0.78–1.04)	0.154
Participants who did not answer and others	70,925	55.26 (53.18–57.31)	1.05 (0.91–1.20)	0.517
Knowledge of households about the healthcare facilities closest to their residence				
Public hospital				
Known	64,817	48.97 (46.47–51.46)		
Not known	65,768	51.03 (48.54–53.53)	0.80 (0.69–0.92)	0.002
Private hospital				
Known	27,836	22.44 (20.32–24.70)		
Not known	102,749	77.56 (75.30–79.68)	0.65 (0.55–0.76)	0.000
Secondary or primary healthcare unit				
Known	116,609	88.92 (87.65–90.08)		
Not known	13,976	11.08 (9.92–12.35)	0.84 (0.70–1.00)	0.051
Clinics or practices of doctors				
Known	32,954	25.7 (23.73–27.77)		
Not known	97,631	74.3 (72.23–76.27)	0.84 (0.73–0.97)	0.019
Midwife practices or maternity hospitals				
Known	18,387	16.59 (14.82–18.52)		
Not known	112,198	83.41 (81.48–85.18)	1.46 (1.24–1.72)	0.000
Integrated health posts (Posyandu)				
Known	56,129	43.23 (41.01–45.47)		
Not known	74,456	56.77 (54.53–58.99)	1.19 (1.06–1.35)	0.004
Village health posts (Poskesdes)				
Known	9932	7.85 (6.63–9.26)		
Not known	120,653	92.15 (90.74–93.37)	1.90 (1.46–2.47)	0.000
Village maternity clinic (Polindes)				
Known	17,312	14.61 (12.95–16.43)		
Not known	113,273	85.39 (83.57–87.05)	1.16 (0.97–1.40)	0.109
Environmental sanitation				
Main water source				
Improved	94,267	72.88 (70.77–74.88)		
Unimproved	36,318	27.12 (25.12–29.23)	1.10 (0.95–1.27)	0.226
Water storage facility				
Improved	127,808	97.56 (96.99–98.03)		
Unimproved	2777	2.44 (1.97–3.01)	1.32 (0.97–1.80)	0.076
Distance from drinking water (time needed to obtain water for drinking)				
Improved	108,053	82.1 (80.44–83.64)		
Unimproved	22,532	17.9 (16.36–19.56)	0.90 (0.77–1.06)	0.218
Wastewater disposal				
Improved	24,099	18.76 (17.35–20.25)		
Unimproved	106,486	81.24 (79.75–82.65)	1.12 (0.98–1.27)	0.089
Slept using a mosquito net				
Yes	63,333	47.44 (45.35–49.54)		
No	67,252	52.56 (50.46–54.65)	1.15 (1.03–1.29)	0.018
Using mosquito coil/electric anti–mosquito mats				
Yes	39,875	31.42 (29.60–33.29)		
No	90,710	68.58 (66.71–70.40)	1.27 (1.13–1.42)	0.000
Covering ventilation holes with anti–mosquito nets				
Yes	8582	6.25 (5.43–7.18)		
No	122,003	93.75 (92.82–94.57)	0.52 (0.43–0.62)	0.000
Using mosquito repellent				
Yes	6562	4.76 (4.18–5.43)		
No	124,023	95.24 (94.57–95.82)	1.06 (0.85–1.31)	0.616
Spraying mosquito spray/insecticide				
Yes	12,004	9.11 (8.10–10.22)		
No	118,581	90.90 (89.78–91.90)	0.66 (0.55–0.79)	0.000
Taking anti–malaria drugs when staying in a malaria endemic area				
Yes	1265	0.92 (0.73–1.16)		
No	129,320	99.08 (98.84–99.27)	0.48 (0.33–0.69)	0.000
Draining the bath water reservoir once a week				
Yes	55,702	41.89 (39.97–43.83)		
No	74,883	58.11 (56.17–60.03)	0.98 (0.87–1.10)	0.698
Settlement or housing condition				
Floors				
Improved	51,788	39.82 (37.95–41.73)		
Unimproved	78,797	60.18 (58.27–62.05)	1.23 (1.08–1.39)	0.001
Walls				
Improved	112,582	85.23 (83.59–86.72)		
Unimproved	18,003	14.77 (13.28–16.41)	1.32 (1.122–1.55)	0.001
Ceiling				
Improved	2192	1.75 (1.45–2.10)		
Unimproved	128,393	98.26 (97.90–98.55)	1.04 (0.72–1.50)	0.835

*lb* Lower 95% confidence boundary of cell percentage, *ub* Upper 95% confidence boundary of cell percentage

^a^95% CI of percentage in univariate analysis

^b^95% CI of percentage in bivariate analysis

### Bivariate analysis

The connections between each explanatory variable and the response variable were analysed with bivariate statistics. The Wald test from logistic regression used a *P* cut-off point of 0.25 because statistical significance may not capture importance and the more traditional levels, such as *P* of 0.05, could fail to select variables known to be essential [13]. A cut-off value of 0.25 is supported by literature [14]. Decisions to keep a variable in the “best” model were based on clinical or statistical significance, or on the significance level of a confounder between 0.1 and 0.15 as it might, in combination with other variables, make an important contribution [13]. In the present study, variables could potentially be entered into the multivariable model if the results of the bivariate test had a value of *P* < 0.25.

### Multivariable analysis

The multivariable analysis aimed to find the parsimonious logistic regression model. A backward technique was used with stepwise removal of non-significant variables (*P* > 0.05). The regression coefficient was repeatedly re-estimated until no further independent variables were insignificant. However, if *P* > 0.05, the variable was inserted into the multivariable model but only if considered substantially necessary. The variables that had significant results in the descriptive analysis of each variable were selected as candidates for the model for multivariable analysis.

## Results

Figure 1 reveals a low prevalence of diagnosed malaria disease at Palu (0.85%) and Donggala (1.56%) districts in Central Sulawesi, and a high malaria prevalence at Intan Jaya (45.96%) and Kepulauan Yapen (38.95%) districts in Papua.

### Descriptive analysis

The effect of social determinants on malaria prevalence in five malaria-endemic provinces of Indonesia is summarised in Table 1 and more detailed in Additional file 2: Appendix S2. A large percentage of participants (72.08%) had not completed high school education, and only 4.19% were considered higher educated. Overall the percentage of males (51.08%) was slightly higher than that of females (48.92%). An OR > 1 shows that the probability of the disease is greater for the response category than the referent category of an explanatory variable. The percentage of respondents who reported “do not know the availability of midwife practices, and village health post” was 83.41% and 92.15%, respectively. In the bivariate analysis, participants who were working were 1.2 times more likely to have malaria than those who were not (OR = 1.20; 95% CI 1.12–1.27; *P* < 0.001). The environmental sanitation variable was not statistically significantly associated with malaria prevalence (OR = 1.13; 95% CI 0.99–1.31; *P* = 0.081). Prevention measures against malaria were important: participants who did not take preventive measures were 1.2 times more likely to contract malaria than those who did (OR = 1.18; 95% CI 1.01–1.38; *P* = 0.036). The risk of having malaria was significantly higher for participants who did not know about the availability of healthcare services (OR = 4.22; 95% CI 1.53–11.59; *P* = 0.005). Further, housing conditions were also important: participants who lived in houses made of unimproved materials were 1.3 times more likely to have malaria than those in houses made of improved building materials (OR = 1.30; 95% CI 1.09–1.54; *P* = 0.003) as shown in Table 2.

**Table 2:** Factors associated with malaria prevalence in the endemic area

Research variables	Simple logistic regression analysis		Multiple logistic regression analysis	
	OR (95% CI)^a^	P-value	AOR (95% CI)^b^	P-value
Gender				
Males (Ref.)				
Females	0.90 (0.85–0.94)	0.000	0.91 (0.87–0.96)	0.000
Age of participants in years				
More than 5 years of age (Ref.)				
Children under 5 years of age	0.72 (0.65–0.81)	0.000	0.74 (0.67–0.83)	0.000
Job (occupation)				
Participants who were not working (Ref.)				
Participants who were working	1.20 (1.12–1.27)	0.000	1.13 (1.06–1.20)	0.000
Use of mosquito nets				
Participants who used mosquito nets (Ref.)				
Participants who did not use mosquito nets	1.09 (0.97–1.23)	0.153	–	–
Knowledge about healthcare services				
Healthcare facilities closest to the residence				
Known (Ref.)				
Not known	4.22 (1.53–11.59)	0.005	4.18 (1.52–11.45)	0.005
Environmental health				
Improved (Ref.)				
Unimproved	1.13 (0.99–1.31)	0.081	–	–
Preventive measures				
Using preventive measures (Ref.)				
Not using preventive measures	1.18 (1.01–1.38)	0.036	–	–
Settlement or housing condition				
Improved (Ref.)				
Unimproved	1.30 (1.09–1.54)	0.003	1.30 (1.09–1.54)	0.003

Ref.: The reference category is represented in the contrast matrix as a row of zeros

^a^Crude odds ratio

^b^Adjusted odds ratio

### Logistic multivariable regression

The OR and AOR of factors affecting malaria prevalence are shown in Table 2 and more detailed in Additional file 2: Appendix S2. The participants who were unaware of the availability of or did not utilize health facilities were more likely to have malaria than those who did (AOR = 4.18; 95% CI 1.52–11.45; *P* = 0.005; adjusted by other covariates). The logistic multivariable regression provides an additional dimension to the research results (Table 2). The final model includes the following significant explanatory variables for malaria prevalence: characteristics of participants (gender, age, and job in block IV), knowledge of the availability of health services (in block V), and settlement (condition of housing structure in block IX).

## Discussion

### Principal findings

Many risk factors increase the likelihood of contracting malaria, particularly the accessibility and utilization of primary healthcare facilities. This study reveals a 4.2-fold increase in the odds of malaria prevalence for participants who do not know about the availability of healthcare facilities compared to those who do know, adjusted by other covariates. The kind of healthcare facilities in this study included government hospitals, private hospitals, primary healthcare (*puskesmas*), clinics, midwife practices, integrated health posts (*posyandu*), village health posts (*poskesdes*), and village maternity clinics (*polindes*). Health services at the primary level in the community as well as their networks are essential for malaria elimination. Healthcare services, particularly for pregnant women, can be delivered during antenatal care (ANC) as pregnant women, infants, and toddlers are especially vulnerable groups for the disease. Malaria is a significant global health issue, especially among pregnant women [15]. Midwives also play a crucial role in health reporting [16]. Although there are physicians and nurses in public and private hospitals, midwives are also needed at the primary level of healthcare and at the community level. Thus, they also need to be equipped with expertise and skills to effectively provide information and promote the prevention of malaria. Particularly at the community level such health promotion and malaria prevention programmes are essential [17]. The findings of this study are consistent with those of one in Uganda where midwives provide malaria-related health promotion and education to pregnant women during every prenatal clinic visit, including direct supervision on how to consume drugs [18]. In sub-Saharan Africa, it has long been recognized that pregnant women are an especially vulnerable group for malaria infection, and that there is a need for active management of the disease in pregnancy as a fundamental part of antenatal care in endemic areas [19]. In Malawi, pregnant women are significant reservoirs of gametocyte transmission which is present in 5% at their first antenatal care visit, and this should not be overlooked in elimination efforts [20].

### Explanatory variables

In the present study, the estimated odds of malaria in females was 10% lower than in males. Similarly, in Lundu district, Sarawak, Malaysia, malaria infection was associated in male than a female with seven-fold risk to be malaria-infected [21]. This is consistent with a previous study showing that females performed a protective function in malaria control [22]. In contrast, in Bungoma county, western Kenya, the risk of clinical malaria was related to being female. As well, *Plasmodium falciparum* infection was connected with being male, poorer, and malnourished [23]. Malaria prevalence differs among age groups. In this study, the estimated odds of malaria for the age group from 35 to 44 years were higher than for others. In a similar study in sub-Saharan Africa, a positive microscopic result was significantly associated with being in the age group of 35–44 years compared to 45 years or older [24]. Also, in South Africa malaria is a significant public health problem among adults and more pronounced in the economically active adult male population [25]. Another study in rural Hausa communities in Nigeria showed that malaria was significantly associated with the participant’s knowledge, age, and gender [26]. In the present study, the risk of having malaria was 1.2 to 1.13 times higher for those who were working (simple logistic and multiple logistic analysis, respectively) compared to those who were not. Conversely, in a study in Blantyre, Malawi, employment status did not differ between the groups [27].

Several other factors are related to malaria prevalence. These include the lack of prevention measures against malaria, such as bed nets, insecticide treatment and knowledge deficits. In spite of a widespread use of mosquito nets at night and insecticide-treated mosquito nets (ITNs), this is not always significantly associated with reduced malaria prevalence. Nevertheless, the present study indicates that participants in endemic provinces of Indonesia who did not use mosquito nets at night were more likely to have malaria than those who did. Similarly, not using ITNs predicted an increased occurrence of clinical malaria in a study in urban Kano, northwestern Nigeria [28], and an Indian study found that a persistent use of nets resulted in a substantial reduction in malaria cases [29]. Illustrating the variability of the relationship between bed-net use and malaria incidence, a study in southern Ethiopia, where the use of bed-nets was frequent, showed that the prevalence of malaria was also high [30]. Obstacles to the use of ITNs include lack of promotion information and lack of knowledge [31]. A survey in Orissa, India, indicated that appropriate communication strategies should be built up and imparted alongside ITN distribution to promote ITN adoption [31]. A similar finding was reported for south-eastern Nigeria where, despite the community having good knowledge about the use of mosquito nets, few knew about the existence of ITNs [32]. Another investigation in Ghana revealed that participants did not have sufficient knowledge about the behaviour of mosquitoes, which weakened their knowledge of the relationship between malaria control and the use of ITNs [33].

Lack of both information and vector control measures to protect people from malaria have been reported as being related to higher malaria risk [34]. Unquestionably, the dissemination of information and health education for preventive measures against malaria are essential. In a South African study, most participants were confident that indoor residual spraying killed mosquitoes and prevented infection. Their sources of malaria information were from the local health facility, radio, and community meetings [35]. The latter study considered that providing health education on malaria and knowledge about risk factors might change health-related behaviour, and thereupon the spreading of knowledge could decrease malaria infection [30]. The present research in the context of Indonesia concludes that preventive measures against malaria in the environment are important.

Knowledge about the availability of health facilities is also important. This study revealed a 4.2-fold increase of malaria prevalence in participants who did not know about the availability of health facilities compared to participants who did. Increasing distance from the place of residence to the nearest health centre was related to delays in seeking treatment for severe malaria at Jinja Hospital, Uganda [30, 36]. In Cambodia, knowledge about malaria symptoms differed significantly between a village with a health centre and an area that had only village malaria workers. Thus, governments need to enhance community knowledge about malaria symptoms and case management in rural areas [37].

Similarly, in sub-Saharan Africa malaria transmission was determined by knowledge of and access to malaria prevention tools as well as healthcare services [38]. In Mali, knowledge and perceptions related to health condition have an important influence on care-seeking behaviour in the formal health sector [39]. The government of Ghana improved access to healthcare, particularly in a primary healthcare programme, and that was an important contribution towards malaria elimination [40]. In the Asia–Pacific region, the use of traditional medicine and/or traditional healers to treat malaria was related to lack of access to health services (due to geographical or economic barriers), belief in traditional medicine, and a perception that symptoms of malaria were less severe a disease [41]. In central Cameroon, rural populations tended to visit traditional practitioners more than urban healthcare providers for geographical and financial reasons [42]. Optimizing the role of the “alert village” where the people of the village can easily access health services through village health posts or other health facilities in the area will reduce malaria risk. The alert village is a strategic effort that was created to accelerate the achievement of the millennium development goals to combat malaria [43]. As noted above, the present study concludes that participants who were unaware of available health facilities were more likely to have malaria than those who did know about these.

Even though environmental sanitation was not significantly associated with malaria prevalence in this study, participants who lived in environments with unimproved sanitation more frequently had malaria than those living in environments with improved sanitation. In a Nigerian study, the majority of respondents believed that bushes around the house were significant facilitators of malaria. Some of them stated that the presence of stagnant water was associated with malaria while others mentioned unclean drainage systems [29]. Keeping the outside environment clean can reduce the risk of malaria as shown in a study in rural Nigeria where reductions of malaria prevalence were significantly associated with periodic cleaning of the external environment [44].

With regards to housing condition, the estimated odds ratio of malaria prevalence for participants who lived in houses made of unimproved materials showed that they were 1.3 times more likely to have malaria than those living in houses made of improved building materials. This is consistent with the results of a study in Nigeria where the odds of malaria infection were significantly higher among participants who lived in unimproved houses [45]. A recent review noted that low-quality housing was consistently associated with malaria prevalence, and the authors recommended that this should be further explored along with housing improvements, especially those that reduce mosquito access [46]. A study in the Ananindeua municipality, State of Pará (Brazil), showed an association between poverty and poor living conditions and highlighted that these need to be considered in malaria prevention and control strategies [47]. Another study, conducted in Equatorial Guinea, showed connections between improved building materials over time, housing quality (closed eaves and door/window screens), and reduced malaria incidence [48]. A study in Krogwe, Tanzania, showed that children living in high-quality housing had only a third of the malaria infections compared to those living in poor quality housing [49]. In addition, location is important with households that are very close to the border of forests and swamps being at high risk for malaria [4, 50]. To sum up, unimproved conditions of housing structure were associated with higher malaria prevalence.

## Limitations of research

Malaria disease status was retrospectively assessed by a standard Riskesdas questionnaire and not directly based on diagnoses made by healthcare professionals. Thus, the prevalence of malaria could only be estimated from respondents who reported that they had been diagnosed with malaria by professional health workers. There may be other factors which affect malaria prevalence but were not monitored in the Riskesdas survey; these could be the subject of further research. Nevertheless, the present study has the strength of being based on a large sample size, and its analyses were novel and robust and identified relationships that could be useful in the future design of malaria control strategies, at least in the five highly endemic provinces of Indonesia.

## Conclusions

This study estimated the socio-demographic factors affecting malaria prevalence in the five highly endemic provinces of Indonesia. These factors included the characteristics of participants, lack of knowledge about the availability of healthcare services, and unimproved housing. Recommendations include increasing community health education regarding the utilization of healthcare facilities, improving community healthcare knowledge, and practices relating to malaria prevention, such as improving the condition of housing structures. These should be considered in upcoming malaria management control strategies.

## Additional files

**Additional file 1: Appendix S1.** Detailed explanation of the scope of variables and analytical method.

**Additional file 2: Appendix S2.** Detailed description of descriptive analysis.

## Abbreviations

ANC: antenatal care
AOR: adjusted odds ratio
API: annual parasite incidence (number of slides positive for parasite × 1000/total population)
ArcGIS: aeronautical reconnaissance coverage geographic information system
Balitbangkes: Badan Penelitian dan Pengembangan Kesehatan (National Institute for Health Research and Development)
CI: confidence interval
HDI: Health Development Index
ITNs: insecticide-treated mosquito nets
MoH: Ministry of Health
OR: odds ratio/unadjusted odds ratio
Polindes: Pos bersalin desa (village maternity clinic)
Poskesdes: Pos kesehatan desa (village health post)
Posyandu: Pos pelayanan terpadu (integrated health post)
Puskesmas: Pusat kesehatan masyarakat (primary health care centre)
Pv: P-values
RDTs: rapid diagnostic tests
Riskesdas: Riset kesehatan dasar (Basic Health Research)
