# Supplementary material for: Social determinants of malaria in an endemic area of Indonesia
Source: Malar J. 2019 Apr 12;18:134. doi: 10.1186/s12936-019-2760-8 (PMC6461805; doi:10.1186/s12936-019-2760-8)
Supplement: Supplementary file 1 — Additional file 1: Appendix S1. Detailed explanation of the scope of variables and analytical method. [file 12936_2019_2760_MOESM1_ESM.pdf]

Appendix S1: Detailed explanation of the scope of variables and analytical method.

Scope of variables

Data handling. Data were processed with Stata software as follows: For the dependent variable (malaria), healthy participants (no malaria) were coded 0 and participants who had malaria were coded 1. Likewise, for the independent variables, a “small” code was given to describe a variable as “good condition” or a “group that is not at risk”. Reference was coded as 0. Stata automatically treated the lowest code in the comparison group as a reference category. These data were collected from questionnaire *RKD 2013.*

Malaria prevalence. In this view, "having malaria" was defined as participants who had ever been recognised as having malaria by health workers. Malaria was characterised as "malaria" or "no malaria" as a binary variable. Health surveyors using a standardised questionnaire collected the data by retrospective assessment. Healthcare professionals asked the participants whether they had ever had a diagnosis of a particular disease (D: Diagnosis). The participants who said that they never had any disease diagnosed were further investigated as to whether they used to/presently experienced certain clinical symptoms of such disease (G: Symptoms). The disease of interest was malaria. Prevalence was measured for one year or less. In the present study, the sample size was 130,585 participants who lived in five out of Indonesia's 33 provinces in 2013. Malaria prevalence in 2013 was 6.0 %. The five provinces with the highest malaria prevalence are: Central Sulawesi, East Nusa Tenggara, Maluku, Papua, and West Papua Provinces (Figure 1).

The characteristics of participants

Gender distinctions were divided into male and female and were taken from questionnaire *b4k4*. The age of the participants was recorded in years; if the age was <1 year "00" was filled in and if the age was ≥97 years "97" was recorded. Age categories were set up and coded as follows: (0) "0 – 4 years"; (1) "5 – 14 years"; (2) "15 – 24 years"; (3) "25 – 34 years"; (4) "35 – 44 years; (5) "45 – 54 years"; (6) "55 – 64 years"; (7) "65 – 74 years"; (8) "more than 75 years ". These data were taken from questionnaire *b4k7*. Education in this paper was defined as the highest level of education attained by participants. Upon completion of high school education, participants were considered as higher educated and coded = 0. Participants who had not completed high school education were seen as low educated and given a code = 1, and, if the respondent was <10 years, the code = 2. These data were collected from questionnaire *b4k8*. For further analysis, the variable 'age of participants' was coded as binary dummy variables with a code = 0 for participants more than five years of age as referent category, and code 1 for less than five years of age. Similarly, for education a code = 0 was given for participants who were considered as higher educated as a referent and a code = 1 was given for others. The primary occupation of participants was taken from questionnaire *b4k9,* and the researcher divided this variable into two groups. If the respondents were not working they were given a code = 0 and if the respondents were working the code = 1.

The behaviour of the participants. From questionnaire *b4k12,* the use of mosquito nets was categorised as follows: If participants slept under mosquito nets at night, these were given a code = 0. If participants did not use mosquito nets, then these were given a code = 1. From questionnaire *b4k13,* the variable of insecticide-treated nets (ITNs) was examined. Those participants sleeping under ITNs to prevent malaria were given a code = 0, while those who did not use ITNs were given a code = 1, and those who did not answer this question were given a code = 2.

Knowledge of health services. Healthcare service access described in the Riskesdas 2013 refers to the knowledge of households about the healthcare facilities nearest to their residence. In this situation, healthcare workers asked the participants about the accessibility and utilisation of healthcare facilities such as a public hospital or government hospitals; private hospitals; primary healthcare centres (puskesmas/pustu); clinics or doctor practices; midwife practices or maternity hospitals; and integrated health posts (posyandu). The participants were also asked regarding village health posts (poskesdes), village maternity clinics (polindes). From the questionnaire, those participants who knew of the availability of the health facilities were given a code = 0, and those who did not know of the availability of the health facilities were given a code = 1. The questionnaire *b5r2k1* shows the availability of government hospitals and *b5r2k1* indicates the availability of private hospitals. Information on primary healthcare centres was obtained from questionnaire *b5r3k1*, information on clinics/practices from *b5r4k1*, and that on midwife practices or maternity hospitals from *b5r5k*. Data about health facilities such as integrated health posts, rural health posts, rural clinics, and traditional health services were obtained from questionnaire *b5r6k1*, *b5r7k1*, *b5r8k1*, and *b6cr1*, respectively. For advanced analysis, participants knowing of the availability of health services were further classified using binary dummy variables with a code = 0 for participants who knew about the availability of certain health facilities and 1 for those who did not know about such health facilities.

The kind of health care facilities and health services in Indonesia such as a public hospital or government hospitals; private hospitals; primary health care centres (puskesmas/pustu); clinics or doctor practices; midwife practices or maternity hospitals or maternity hospitals; and integrated health posts (posyandu), village health posts (poskesdes), village maternity, and village clinics (polindes) in generally. (Heywood & Harahap, 2009; Mahendradhata et al., 2017)

Environmental sanitation. Environmental sanitation included information on the primary source of water, distance to drinking water, and wastewater disposal. According to questionnaire *b8r1a*, participants who had improved drinking water were given the code = 0, and those who did not the code = 1.

The questionnaire variable *b8r1a* consist of the main clean water supply of household. This variable categorised improved when the participants use water taps, buying water from water taps, drilled well pump, well water sheltered, the water spring protected, and rainwater storage. Contrarily, the variable categorised unimproved for who use well water is not protected, the water spring is not protected, and water from the river, lake, and irrigation.

According to questionnaire *b8r3c,* participants who had an improved primary source of water were given a code = 0, and a code = 1 if it was not improved. The questionnaire variable *b8r3c* consist of the drinking water storage. This variable categorised improved when the participants drinking water storage from the dispenser, kettle, thermos, and jerry cans, kind of earthenware jug and bucket, covered pans. In another way; the variable categorised unimproved for who drinking water storage from the bucket, and open pans.

The same categories were coded for participants who responded to drinking water needs in questionnaire *b8r6a*. The questionnaire *b8r6a* is the distance which needed to drinking water needs. This variable categorised improved for participants who get drinking water where the location of the drinking water in the house, the distance to get drinking water needs is less than or equal to 100 meters. Differently, this variable categorised unimproved for participants who get drinking water where the range of drinking water between 101-1,000 meters and more than 1,000 meters. Wastewater disposal was for those participants who managed domestic wastewater disposal from water taps, kitchens, and bathing areas in questionnaire *b8r10.* Further, for bivariate and multivariable analysis, the environmental sanitation variable was composited into binary dummy variables with a code = 0 for participants whose environmental sanitation was improved and a code = 1 for those with unimproved

environmental sanitation. Similar codes were given for the variable of settlement or housing condition of the participants of the study.

Behaviour to prevent mosquito bites. These independent variables were selected from questionnaire *b8r14*. According to questionnaire *b8r14a*, if participants slept using mosquito nets they were coded = 0, and if not, = 1. The same categories were coded for participants who used mosquito coils, and/or electric mosquito repellents in questionnaire *b8r14b*. Similar coding was used for participants who covered ventilation holes with anti-mosquito nets in questionnaire *b8r14c*; participants who used mosquito repellent to avoid mosquito bites in questionnaire *b8r14d*; participants who used spray with mosquito insecticide in questionnaire *b8r14e*; participants seeking anti-malarial drugs for malaria prevention when staying in a malaria endemic area in questionnaire *b8r14f*. Furthermore, behaviours preventing mosquito bites were composited into binary dummy variables with a code = 0 for participants who took prevention measures, and a code = 1 for those who had not.

Housing condition. Questionnaires *b9r4*, *b9r5*, and *b9r6* regarding “the widest type of tile”, “the widest type of wall”, and “the widest type of ceiling”, respectively, describe conditions of houses inhabited by the participants. Participants who had the kind of housing conditions considered "improved”, were given the code = 0 and the others were given the code = 1.

In this study, the settlement or housing condition is a composite of variables: floors, walls, and ceiling which categorised improved and unimproved. Improved flooring is categorised who those use the kind of the widest floor of housekeeping with ceramics, tiles, marble, and cement floor. Contrarily, unimproved flooring who use the widest floor with cement plastering cracked, boards, bamboo, wicker bamboo, and rattan, and soil. Further, improved wall who those use the kind of the wall of housekeeping with stonewall panels and wood, board, and or plywood. On the contrary, unimproved wall who use the widest wall with

bamboo, zinc wall. Also, the variable improved ceiling categorised who use the kind of the widest ceiling of housekeeping with concrete and gypsum. Conversely, an unimproved wall categorised for participants who use the kind of the widest ceiling of housekeeping: asbestos and GRC board wood and or plywood, woven bamboo or nothing. The criteria environmental health of material houses are based on joint monitoring programme WHO-UNICEF in Riskesdas 2013.

Details of data analysis

Data were analysed using the statistical data processing applications by Stata taking into account the complex sampling design (David W. Hosmer, 2013). Data included the proportion of participants with malaria, the characteristics of participants, the behaviour of participants, the accessibility and utilisation of health services, environmental sanitation, mosquito bite prevention measures, and housing conditions. These data were analysed using Stata 14. In univariate analysis we used the command "svy: tabulate" for one-way tabulations for complex survey data. The primary characteristic is that “svy: tabulate” computes a standard of independence that is useful for complex survey data. Parameter confidence intervals and standard errors can optionally be displayed for weighted counts or row, cell, or column proportions. Furthermore, the 95% CI for proportions are set up using a logit transform so that their endpoints always lie between 0 and 1.

Social data analysis commonly uses multivariable regression. In multivariable regression, explanatory variables do not come into the regression simultaneously but step by step according to p-value. The variable which has the largest *p-value* is the first to be removed from the model. The model was retested again to evaluate the effect of the deletion of one variable which had a p-value > 0.05, and it was found to have no confounding effect. As a rule of thumb, if the regression coefficient from the simple regression model changes by more

than 10%, then an independent (predictor or explanatory) variable is said to be a confounder. Simple logistic regression analysis refers to the regression application with one dichotomous outcome that is malaria prevalence and one independent variable. At this stage, we show crude odds ratios (OR) with 95% CI. In bivariate analysis, some of the variables with a p-value > 0.05 were still inserted into the multivariable model but only when these variables were considered substantially necessary. Multiple logistic regression analysis applies when there is a single dichotomous outcome and more than one independent variable. It will be referred to as "multivariable analysis". At this stage the adjusted odds ratio (AOR) in 95% CI is shown. In the multivariable analysis, we selected only variables with a p-value < 0.05 as presented in table 2.

In multiple regression situations, scientists are affected by working out the "strongest" predictors in the analysis. Logistic regression requires a categorical dependent variable. By-passing bivariate logistic regressions, independent variables that may have predictive value for the dependent variable were selected for the multiple regression models (Wald test, ***P*** < 0.25) (Bursac, Gauss, Williams, & Hosmer, 2008).

References

Bursac, Z., Gauss, C. H., Williams, D. K., & Hosmer, D. W. (2008). Purposeful selection of variables in logistic regression. *Source Code for Biology and Medicine, 3*(1), 17. doi:10.1186/1751-0473-3-17

David W. Hosmer, J. S. L., Rodney X. Sturdivant. (2013). *Applied Logistic Regression, Third Edition*: Wiley Series in Probability and Statistics.

Heywood, P., & Harahap, N. P. (2009). Health facilities at the district level in Indonesia. *Australia and New Zealand Health Policy, 6*(1), 13.

Mahendradhata, Y., Trisnantoro, L., Listyadewi, S., Soewondo, P., Marthias, T., Harimurti, P., & Prawira, J. (2017). The Republic of Indonesia health system review.
