# Supplementary material for: Social determinants of malaria in an endemic area of Indonesia
Source: Malar J. 2019 Apr 12;18:134. doi: 10.1186/s12936-019-2760-8 (PMC6461805; doi:10.1186/s12936-019-2760-8)
Supplement: Supplementary file 2 — Additional file 2: Appendix S2. Detailed description of descriptive analysis. [file 12936_2019_2760_MOESM2_ESM.docx]

## Appendix S2: Detailed description of data analysis.

This study uses syntax survey both “svy: tabulate” and “svy: logistic” to show the effect of the social determinants of malaria in an endemic area in 5 *P*rovinces of Indonesia. Descriptions of variables appear in table 1 in the text. The univariate and bivariate analysis of baseline socio-demographic characteristics of study participants used a complex design. In this case, it provided basic data and health status indications as well as health contributing factors at household and individual level (characteristics of participants) covering knowledge of health access and utilisation of health services; prevention measures against malaria; environmental health; and settlement.

## Descriptive analysis

*Characteristics of participants.* The percentage of male (51.08%) was slightly higher than the percentage of females (48.92%), while the percentage of participants who fell in the age range of 5-14 years was the highest (26.06%). Furthermore, a significant percentage of participants had not completed high school education (72.08%) and only 4.19% had a higher education is with others at 23.72%. For employment, the percentage of participants not working was 60.12% compared to 39.88 % who do work.

*The behaviour of the participants.* A higher percentage of participants did not use mosquito nets (53.81%), and the majority of participants did not answer concerning insecticide-treated nets (55.26%) given that only 23.26% claimed to use insecticide treated bed-nets (ITNs).

*The accessibility and utilisation of health service.* The general a knowledge of health access and utilise to healthcare facilities was found to be no good. *P*articipants who thought they did not have knowledge of health access and so did not utilise health services at the public hospital were 51.03%, private hospital (77.56%) and clinics or doctor practices (74.30%), integrated health post (posyandu) (56.77%), village health post (poskesdes) (92.15%), village maternity clinic (polindes) (85.39%) respectively. Also, this study concludes with 95% confidence that the number of people in the population that fall within the category that they did not know the availability or did not utilise midwife practices or maternity hospitals category was 83.41% (95% CI 81.48-85.18). Otherwise, those who have knowledge of access and utilise primary health care (*P*HC) were 88.92% (95% CI 87.65-90.08) which is good compared with other healthcare facilities.

*Environmental sanitation.*

The scope of water in the Riskesdas 2013 report includes the type of water source for household and drinking purposes. The proportion of households with access to improved water sources in the study areas was 72.88%. Environmental sanitation such as a facility to keep the water (97.56%), and distance to drinking water (82.1%) have overall improved. In contrary, those who have not improved wastewater disposal comprised 81.24 % (95% CI 79.75 - 82.65)

*Behaviour preventing mosquito bites*. *P*articipants asked what they usually do to prevent disease due to mosquito bites. The question regarding a. Using mosquito net; b. Using mosquito coil/electric anti-mosquito mats; c. Covering ventilation with anti-mosquito nets; d. Using mosquito repellent; e. spraying mosquito spray/insecticide, and f. taking anti-malaria drugs when staying at endemic malaria area. In general, participants who have behaviour to self-prevent from mosquito bites still less. It is essential to protect participants from mosquitoes not just to prevent annoying itchy bites, but to stop the spread of diseases that the mosquito can carry. The percentage who did not sleep using mosquito net was 52.56%, and who did not use toxic mosquito was 68.58%. Those who did not use mosquito netting to cover ventilation were 93.75% and who did not use mosquito repellent 95.24%. Those that did not spray mosquito insecticide was 90.9%, and did not take anti-malarial drugs was 99.08%.

*Housing condition*. In common, those who have a material building that was not improved such as flooring (60.18%) and ceiling (98.26%). In contrast, those with improved walls comprised 85.23%.

## Bivariate analysis

*Characteristics of participants.* Malaria prevalence differed by gender and age groups. Estimated odds of malaria prevalence in females is 10% lower than males (OR = 0.90; 95% ; CI = 0.85 - 0.94; *P* < 0.001). It would mean that males have 1.11 times higher odds of malaria prevalence compared to females.

Similarly, the OR estimates of malaria prevalence in children under five years of age are 1.39% less likely to have malaria prevalence than the participants who have more than five years age (OR = 0.72; 95% CI = 0.65 - 0.81; *P* < 0.001). Besides, this study obtains an OR for malaria prevalence of 1.2 times for participants who were working versus those who were not working. It means that the odds of malaria prevalence are 1.2 times as high (or 20% higher) for those who were working than those who were not working.

*The behaviour of the participants.* According to the behaviour of the participants, use mosquito nets, and insecticide-treated nets were not significantly related to the prevalence of malaria. However, the *P*articipant did not use mosquito nets at night more likely to have malaria than those who did (OR = 1.09; 95% CI (0.97 - 1.23); *P* = 0.153)

*Knowledge of households about the nearest healthcare facilities to their residence. P*articipants who do not know the availability of or do not utilise healthcare are more likely to have malaria. Estimated odds of malaria prevalence in participants who not know the availability of health facilities and health service is 4.2 times more than participants who know the availability of health facilities (OR = 4.22; 95% CI = 1.53 - 11.59; *P* = 0.005).

*Environmental sanitation.* In general, participants who do not have improved environmental sanitation more likely to have malaria 1.1 times than those have unimproved environmental sanitation with (OR = 1.13; 95% CI (0.99 - 1.31); *P* = 0.081). In this study, the *P* of the variable was < 0.25. The Wald test from logistic regression used a *P* cut-off point of 0.25 because significance may not capture the importance and the more traditional levels, such as *P* of 0.05 can fail to select variables known to be essential.

*Behaviour preventing mosquito bites.* In general, the risk of contracting malaria in participants who had no behaviour to prevent mosquito bites have a higher risk of contracting malaria. Based on the composite variable of this variable, the research reveals that participants who did not take preventive measures from biting mosquitoes were 1.2 times more likely to contract malaria than those who did (OR = 1.18; 95% CI (1.01 - 1.38); *P* = 0.036).

*Housing condition*. With regards to housing condition, estimated odds of malaria, participants who live in houses composed of unimproved materials were 1.3 times more likely to have malaria than those living in houses composed of improved building materials (OR = 1.30; 95% CI (1.09 -1.54); *P* = 0.003)
